# Transcriptomic and phytochemical analysis of the biosynthesis of characteristic constituents in tea (*Camellia sinensis*) compared with oil tea (*Camellia oleifera*)

**DOI:** 10.1186/s12870-015-0574-6

**Published:** 2015-08-07

**Authors:** Yuling Tai, Chaoling Wei, Hua Yang, Liang Zhang, Qi Chen, Weiwei Deng, Shu Wei, Jing Zhang, Congbing Fang, Chitang Ho, Xiaochun Wan

**Affiliations:** State Key Laboratory of Tea Plant Biology and Utilization, Anhui Agricultural University, Hefei, 230036 People’s Republic of China; Department of Food Science, Rutgers University, Rutgers, NJ USA

**Keywords:** Comparative transcriptome, *Camellia sinensis*, *Camellia oleifera*, Chemical constituents, Metabolic pathways

## Abstract

**Background:**

Tea plants (*Camellia sinensis*) are used to produce one of the most important beverages worldwide. The nutritional value and healthful properties of tea are closely related to the large amounts of three major characteristic constituents including polyphenols (mainly catechins), theanine and caffeine. Although oil tea (*Camellia oleifera*) belongs to the genus *Camellia*, this plant lacks these three characteristic constituents. Comparative analysis of tea and oil tea via RNA-Seq would help uncover the genetic components underlying the biosynthesis of characteristic metabolites in tea.

**Results:**

We found that 3,787 and 3,359 bud genes, as well as 4,042 and 3,302 leaf genes, were up-regulated in tea and oil tea, respectively. High-performance liquid chromatography (HPLC) analysis revealed high levels of all types of catechins, theanine and caffeine in tea compared to those in oil tea. Activation of the genes involved in the biosynthesis of these characteristic compounds was detected by RNA-Seq analysis. In particular, genes encoding enzymes involved in flavonoid, theanine and caffeine pathways exhibited considerably different expression levels in tea compared to oil tea, which were also confirmed by quantitative RT-PCR (qRT-PCR).

**Conclusion:**

We assembled 81,826 and 78,863 unigenes for tea and oil tea, respectively, based on their differences at the transcriptomic level. A potential connection was observed between gene expression and content variation for catechins, theanine and caffeine in tea and oil tea. The results demonstrated that the metabolism was activated during the accumulation of characteristic metabolites in tea, which were present at low levels in oil tea. From the molecular biological perspective, our comparison of the transcriptomes and related metabolites revealed differential regulatory mechanisms underlying secondary metabolic pathways in tea versus oil tea.

**Electronic supplementary material:**

The online version of this article (doi:10.1186/s12870-015-0574-6) contains supplementary material, which is available to authorized users.

## Background

Tea is produced from the plant *Camellia sinensis* (L.) O. Kuntze in the family *Theaceae*. Tea is one of the most popular beverages worldwide, and tea leaves represent an important source of many biologically active metabolites such as flavonoids, theanine and caffeine [1, 2]. Flavonoids mainly comprise flavan-3-ols (catechins), epicatechin (EC), gallocatechin (GC), epigallocatechin (EGC), catechin (C) and their respective gallate esters, such as epigallocatechin gallate (EGCG) and epicatechin gallate (ECG) [3]. Tea leaves, which contain various secondary metabolites, are usually used as the raw material for tea production. However, the molecular mechanisms that regulate the biosynthesis of catechins, theanine and caffeine in tea remain elusive.

Great effort has focused on elucidating the molecular mechanisms underlying plant growth, development [4, 5] and secondary metabolite production [6] in tea. Most of these studies have focused on characterizing genes related to secondary metabolism, most of which were revealed through EST sequencing [7] and analysis of the transcriptomes from various tissues of tea plants [8, 9] or under different stress conditions [10, 11]. More recently, Shi *et al.* discovered novel candidate genes involved in pathways in tea by analyzing transcriptome data [12]. Liu *et al.* reported the discovery of a novel enzyme involved in galloylated catechin biosynthesis in tea plants [13]. However, the lack of genomic information has become an obstacle to exploring the molecular mechanisms underlying secondary metabolite biosynthesis in tea. Transcriptome sequencing represents an efficient approach to obtaining functional genomic information.

RNA-Seq is a rapid technique for genome-wide gene expression analysis that is widely used to determine gene structures and expression profiles in model organisms. *De novo* assembly of RNA-Seq data makes it possible to conduct gene analysis in the absence of reference genomes [14–16]. Comparative transcriptomic studies have been performed to identify differential gene expression in several organisms [17–20].

Another widely known member of *Theaceae* is oil tea, *Camellia oleifera* Abel, a tree serving as an important source of edible oil that is grown specifically in China. Oil tea was genetically closely to tea, and they both belonged to genus *Camellia*. Here, we performed RNA-Seq on buds and second leaves of tea and oil tea to characterize differences in gene expression between these two plants. This comparative transcriptomic analysis provides important insights into the molecular mechanisms underlying secondary metabolite biosynthesis in tea, as well as the phytochemical characteristics of its main metabolites.

## Results

### Analysis of the contents of catechins, theanine and caffeine

**Fig. 1:**
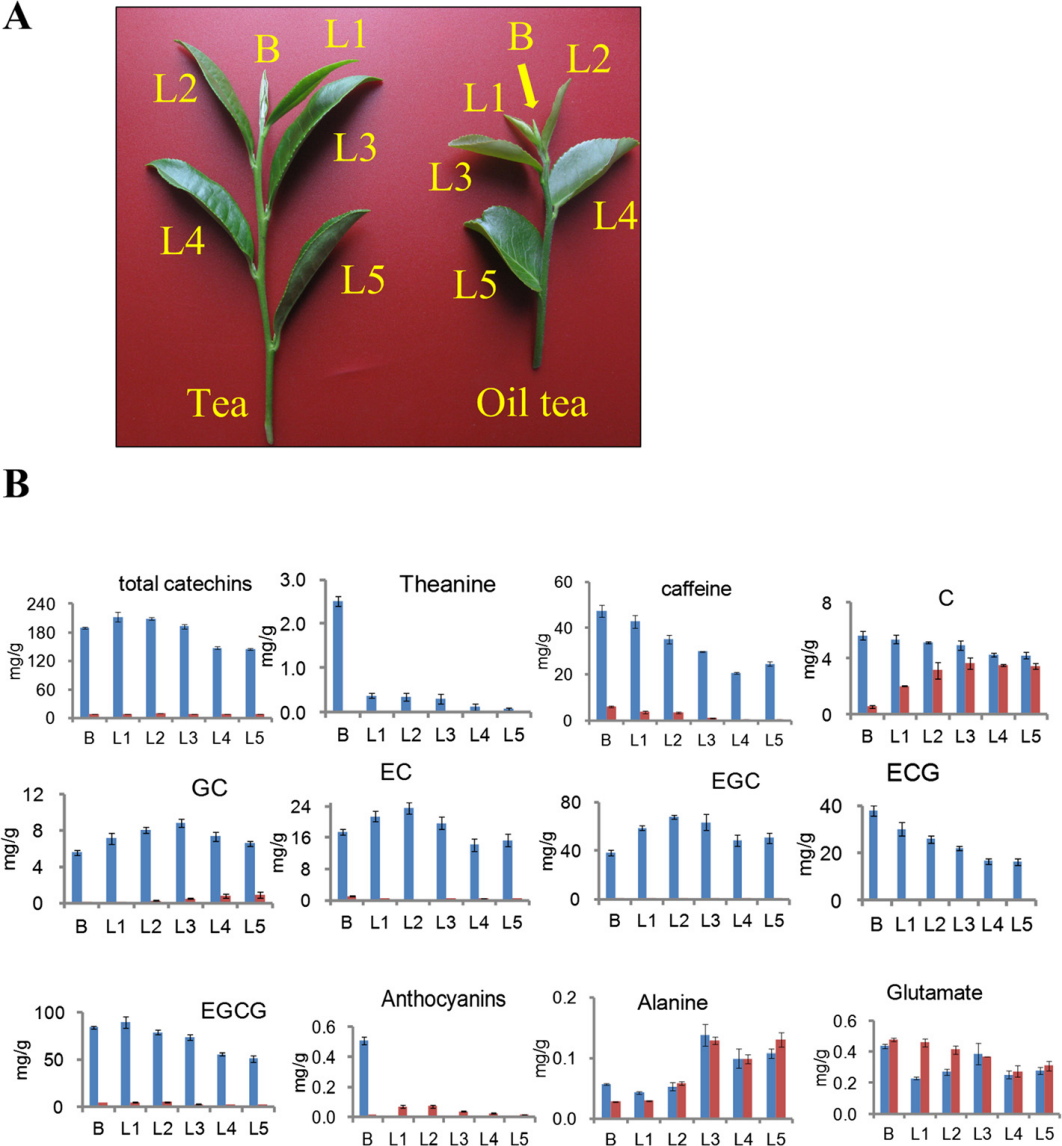
Determined contents of compounds involved in three main secondary metabolic pathways. **a** Samples examined. Buds and five initial leaves were collected from tea and oil tea. Bud, L1, L2, L3, L4 and L5 indicate the bud, first, second, third, fourth and fifth leaf, respectively. **b** Variation in the contents of compounds biosynthesized by catechin-, theanine- and caffeine-related metabolic pathways

HPLC analyses were conducted to determine the contents of catechins, theanine and caffeine, and related intermediates in buds and five leaves of tea and oil tea (Fig. 1). All standard compounds showed good linearity (R^2^ > 0.9991) in a relatively wide concentration range. Compared to oil tea, most of these metabolites were present at higher concentrations in tea (Fig. 1b). The average contents of three characteristic components (total catechins, theanine and caffeine) in tea leaves were 1.5- to 173-fold higher than those in oil tea leaves. In particular, tea contained over a 180 mg/g of total catechins in its leaves and buds. The only exception is that the anthocyanin content in oil tea leaves was 32-fold higher than that in tea leaves. These results confirm that tea is rich in catechins, theanine and caffeine (Table 1).

**Table 1:** Variation in the contents of major compounds in tea and oil tea

Compounds	Content in buds (mg/g)		Tea bud/Oil tea bud	Average content in leaves (mg/g)^a^		Tea leaves/Oil tea leaves
	Tea	Oil tea		Tea	Oil tea	
Total catechins	189.00	7.63	24.8	181.00	8.76	20.7
Theanine	2.50	0.01	252.5	0.25	0.00	86.8
Caffeine	47.50	5.98	7.9	30.50	1.81	16.8
C	5.65	0.60	9.4	4.79	3.18	1.5
GC	5.54	0.00	76,654.6	7.57	0.51	14.8
EC	17.40	1.12	1.1	18.90	0.43	43.8
EGC	38.20	0.38	12.5	57.70	0.33	173.3
ECG	0.52	13.20	2.9	22.10	0.52	42.9
EGCG	4.98	4.98	16.9	69.90	3.75	18.7
Anthocyanins	0.51	0.02	32.5	0.00	0.04	0.0
Alanine	0.06	0.03	2.04	0.08	0.09	0.96
Glutamate	0.44	0.44	1.0	0.29	0.37	0.8

^a^ Average contents in leaves were calculated using values from the five initial leaves

Moreover, the contents of these characteristic constituents varied during the period from the appearance of buds to the appearance of the five leaves. The levels of GC, EGC and EC increased from the bud to the second or third leaves in tea, whereas a general decline in caffeine, total catechins, ECG and EGCG levels was observed in tea leaves.

A steady decrease in theanine levels was observed from the first leaf to the fifth leaf in tea, and the levels of this compound were almost seven-fold greater in buds than in leaves. A similar variation was detected in oil tea, but the absolute contents were much lower. Due to the variation in the contents of most compounds (EC, EGC, GC and ECG) in the three initial leaves, we selected the second leaves and buds of tea and oil tea for RNA-Seq.

### *De novo* assembly and comparative analyses of RNA-Seq data

We utilized Illumina RNA-Seq technology to sequence the buds and second leaves of tea and oil tea. After removing adaptor sequences, duplication sequences, ambiguous reads and low-quality reads, a total of 23.4 Gb of clean reads was generated, with an average of 5.85 Gb clean reads per sample (Table 2).

**Table 2:** Statistics from the generated RNA-Seq reads

	Sample	Total raw reads	Total clean reads	Total clean nucleotides (nt)	Q20 percentage	N percentage	GC percentage
Tea	buds	78,077,028	66,059,720	5,945,374,800	97.50 %	0.01 %	46.71 %
	leaves	71,399,954	65,258,822	5,873,293,980	97.36 %	0.01 %	46.34 %
Oil tea	buds	86,743,714	65,259,264	5,873,333,760	97.44 %	0.01 %	46.57 %
	leaves	73,366,916	63,928,844	5,753,595,960	97.50 %	0.01 %	46.76 %

The final assembly of tea had 81,826 unigenes with an N50 length of 1,265 bp (Table 3). Functional annotation revealed 53,786, 49,174, 34,636, 31,024, 18,748 and 40,838 unigenes with alignments to the NR (Non-redundant protein database), NT (Non-redundant nucleotide database), Swiss-Prot (Annotated protein sequence database), KEGG (Kyoto encyclopedia of genes and genomes), COG (Clusters of orthologous groups of protein) and GO (Gene ontology) databases, respectively. The final assembly of oil tea consisted of 78,863 unigenes with an N50 length of 1,254 bp. Of these, 54,115, 49,009, 34,682, 30,990, 19,126 and 41,325 unigenes were annotated by alignment against the NR, NT, Swiss-Prot, KEGG, COG and GO databases, respectively (Additional file 1). Sequence comparisons revealed that 17,459 genes are shared by both tea and oil tea, 9,725 of which were mapped to KEGG pathways (http://www.genome.jp/kegg/) [21]. High amino acid sequence identity was found in the homologous genes between tea and oil tea, as 64 % of the genes shared over 70 % identity. We also detected 64,826 specific transcripts in tea and 61,863 in oil tea.

**Table 3:** Summary of assemblies of RNA-Seq data

Species		Sample	Total number	Total length(nt)	Mean length(nt)	N50 length(nt)	Total consensus sequences	Distinct clusters	Distinct singletons
Tea	Contig	buds	157,832	50,217,182	318	508	-	-	-
		2nd leaves	151,557	47,978,363	317	507	-	-	-
	Unigene	buds	89,155	55,909,930	627	1103	89,155	30,384	
Oil tea		2nd leaves	83,415	83,415	53,056,344	636	1099	83,415	28,571
	All unigenes		81,826	64,132,659	784	1265	81,826	33,079	48,747
	Contig	buds	153,407	47,519,556	310	491	-	-	-
		2nd leaves	148,760	47,208,814	317	508	-	-	-
	Unigene	buds	84,244	52,523,964	623	1073	84,244	30,379	
		2nd leaves	83,679	53,084,367	634	1097	83,679	29,963	
	All unigenes		78,863	62,035,893	787	1254	78,863	34,041	44,822

### Analysis of the differentially expressed genes (DEGs)

**Fig. 2:**
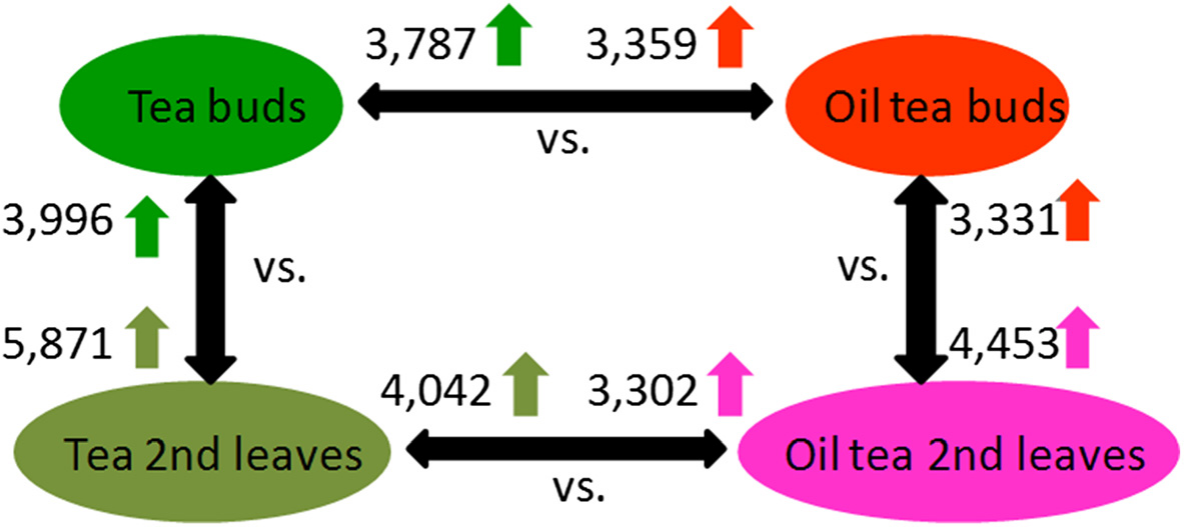
Number of identified DEGs identified by comparing gene expression levels between any two tissues. The numbers of significantly up-regulated genes (log2(Fold-change) > 1; FDR < 0.001) between two tissues (indicated by double-headed arrows). Arrows indicate up-regulated genes associated with each number

**Fig. 3:**
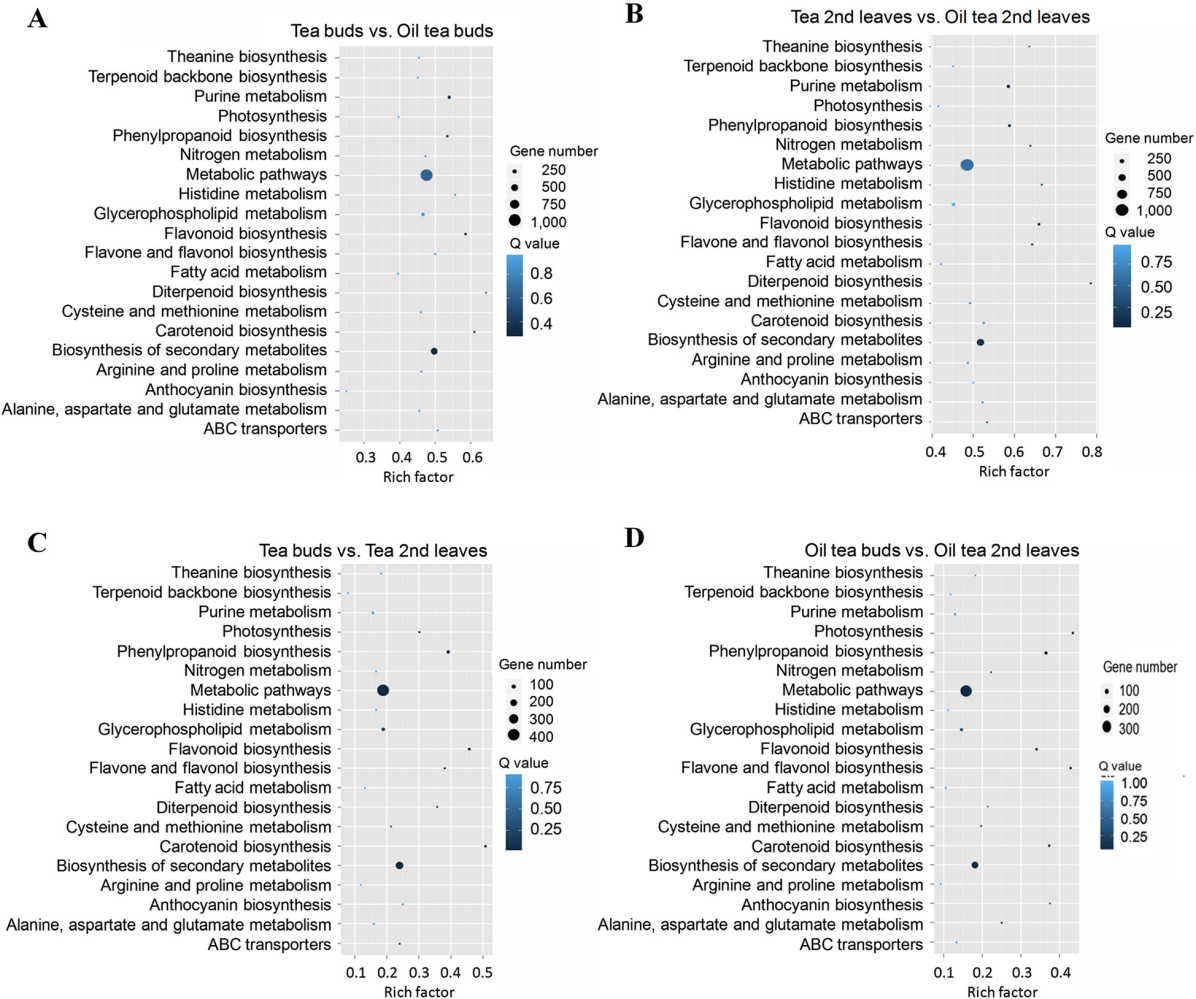
Pathway enrichment analysis involving comparisons between any two tissues. The rich factors indicate the ratio of the number of DEGs mapped to a certain pathway to the total number of genes mapped to this pathway. Greater rich factor means greater intensiveness. The Q value was calculated using hypergeometric test through Bonferroni Correction. Q value is coorrected p value ranging from 0-1, and less Q value means greater intensiveness. Gene number means number of DEGs mapped to a certain pathway

The DEGs were identified by comparing FPKM (Fragment Per Kilobase of exon model per Million mapped reads) values [22] between different libraries under the thresholds of log2 (Fold-change) over 1 and FDR less than 0.001 (Fig. 2 and Additional file 2). The results indicated that both tea and oil tea had more genes with higher transcription levels in the second leaves than in buds. Compared with oil tea, tea contained more DEGs (3,787 in buds and 4,042 in leaves) with increased expression in both buds and leaves than oil tea (3,359 in buds and 3,302 in leaves). Next, we analyzed the DEGs using KEGG pathway analysis, which assigned 4,226 DEGs derived from tea buds versus oil tea buds (TBvsOTB), 4,174 from tea buds versus tea leaves (TBvsTL), 4,334 from tea leaves versus oil tea leaves (TLvsOTL) and 3,418 from oil tea buds versus leaves (OTBvsOTL). High proportions of these DEGs are involved in secondary metabolite pathways, including 483 DEGs (11.43 %) from TBvsOTB, 503 (11.61 %) from TL2vsOTL2, 594 (14.23 %) from TBvsTL and 482 (14.1 %) from OTBvsOTL, respectively. The estimated rich factors (number of DEGs mapped to a certain pathway/total number of genes mapped to this pathway) of secondary metabolism were 0.4–0.7 in TBvsOTB and TLvsOTL (Fig. 3a and b), whereas they were 0.1–0.3 in TBvsTL and OTBvsOTL (Fig. 3c and d). The DEGs identified through comparisons between tea and oil tea were clustered in the pathway secondary metabolism, suggesting that there are different secondary metabolism pathways in these two species. A lower rich factor between two stages for either of two species implies that steady metabolism occurs during this period (Additional file 3).

Based on alignments against the Swiss-Prot, COG and KEGG databases with an e-value cutoff of less than 1 × 10^−30^, 117, 51 and 18 tea genes and 110, 52 and 20 oil tea genes were found to be involved in the biosynthesis of catechins, theanine and caffeine, respectively (Additional file 4). We detected over 200 homologous genes in tea and oil tea encoding enzymes potentially involved in catalyzing these reactions. Tea and oil tea contain a similar number of genes encoding most enzymes in the assembled gene models, but their transcription levels are considerably different (Table 4).

**Table 4:** Transcription levels of genes involved in the biosynthesis of catechins, theanine and caffeine

Pathway	Enzyme		Gene #	Tea buds	Tea leaves	Gene #	Oil tea buds	Oil tea leaves	Max.Log10(Tea FPKM)-Max. Log10(Oil Tea FPKM	
	Description	Abbr.		Max. Log10 (FPKM)	Max. Log10 (FPKM)		Max. Log10 (FPKM)	Max. Log10 (FPKM)	Bud	2nd leaf
Catechines	phenylalanine ammonia-lyase	PAL	21	6.12	5.71	20	5.22	5.01	0.90	0.70
	cinnamic acid 4-hydroxylase	C4H	_	_	_	_	_	_	_	_
	4-coumarate--CoA ligase	4CL	16	5.60	5.61	17	5.60	5.82	0.00	−0.21
	chalcone synthase	CHS	37	7.27	6.90	36	7.59	7.22	−0.32	−0.32
	chalcone isomerase	CHI	1	5.34	4.83	1	5.68	5.27	−0.34	−0.44
	flavanone 3-hydroxylase	F3H	5	7.18	6.61	6	5.70	5.66	1.48	0.95
	flavonoid 3',5'-hydroxylase	F3'5'H	5	6.10	5.75	4	5.40	4.90	0.70	0.85
	flavonoid 3'-hydroxylase	F3'H	_	_	_	_	_	_	_	_
	leucoanthocyanidin reductase	LAR	4	6.93	7.15	6	5.10	6.33	1.83	0.82
	anthocyanidin synthase	ANS	_	_	_	_	_	_	_	_
	anthocyanidin reductase	ANR	5	6.97	6.46	2	6.55	6.09	0.42	0.37
	flavone synthase	FNS	_	_	_	_	_	_	_	_
	dihydroflavonol 4-reductase	DFR	11	4.10	3.79	10	3.68	3.52	0.42	0.27
	flavonol synthase	FLS	12	3.43	3.14	8	4.08	4.23	−0.65	−1.09
Theanine	glutamate synthase	GOGAT	36	3.43	3.73	34	3.95	3.98	−0.52	−0.25
	glutamate dehydrogenase	GDH	9	2.83	2.55	8	2.87	3.12	−0.04	−0.57
	alanine aminotransferase	ALT	1	3.47	3.05	3	0.00	0.00	3.47	3.05
	glutamine synthetase	GS	5	6.54	5.76	7	5.72	6.18	0.82	−0.42
	theanine synthetase	TS	_	_	_	_	_	_	_	_
Caffeine	5'-nucleotidase	5'-Nase	4	3.95	3.60	5	4.12	3.76	−0.17	−0.16
	IMP dehydrogenase	IMPDH	1	5.26	4.95	1	3.83	3.69	1.43	1.26
	ribokinase	RBK	11	4.08	4.24	13	5.18	5.02	−1.10	−0.78
	caffeine synthase	TCS	2	6.63	6.34	1	2.56	2.49	4.07	3.85

### Identification of DGEs involving in characteristic metabolic pathways in tea

**Fig. 4:**
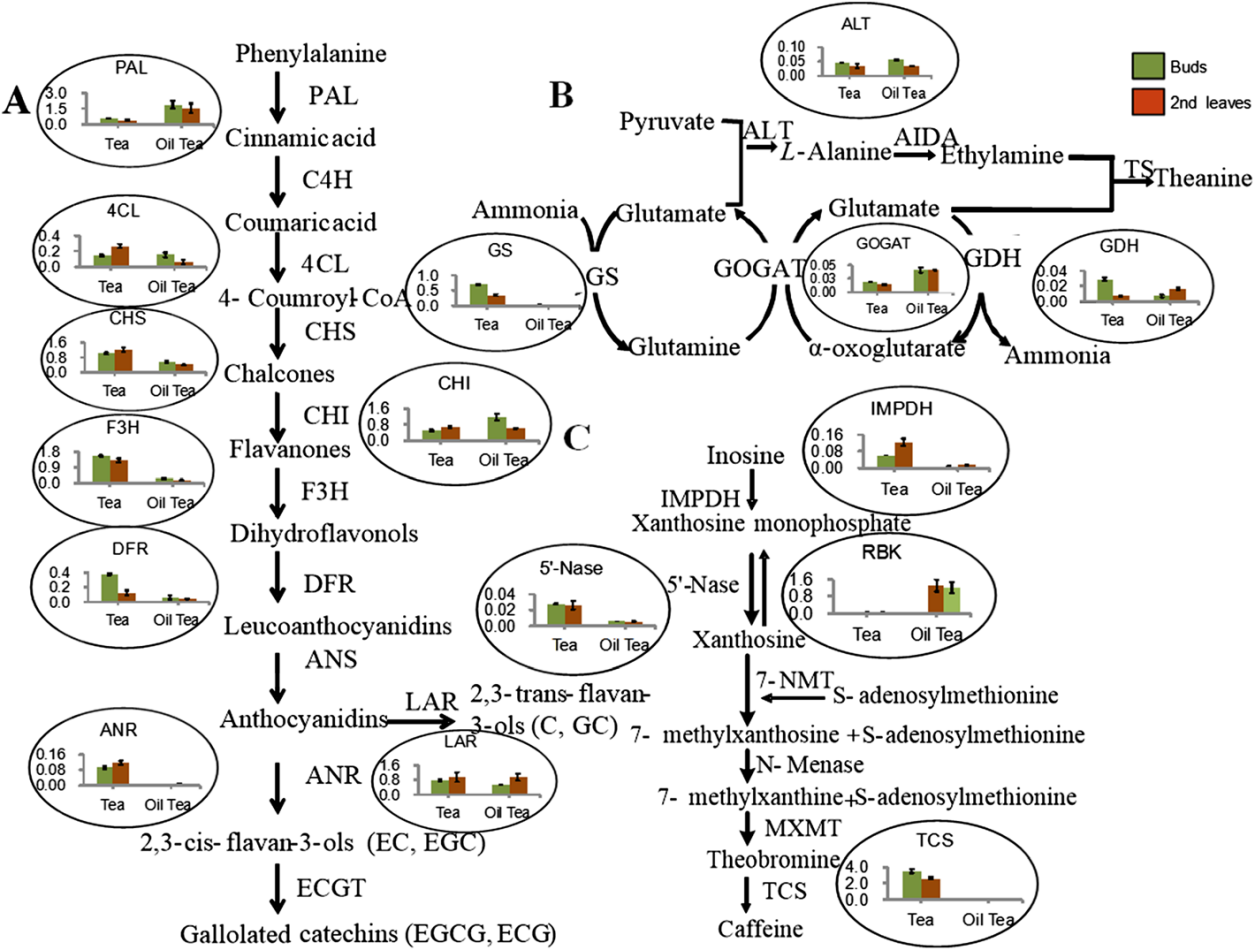
Pathways of the three main secondary metabolites in tea and oil tea. **a** Core reactions of flavonoid biosynthesis involved in the biosynthesis of catechins. Compound names are shown below each arrow. Abbreviations beside the arrows indicate the enzymes catalyzing the transfer. The gene expression levels detected by qRT-PCR are shown in the histograms within the corresponding circles. Transcription level is indicated as the mean (2^ΔCt^) ± SD. **b** Core reactions of the theanine biosynthesis pathway. (C) Core reactions of the caffeine biosynthesis pathway

We used qRT-PCR to confirm the differential expression levels of 34 DEGs involved in the biosynthesis of catechins, theanine and caffeine and quantified their maximum transcription levels in tea and oil tea (Fig. 4 and Additional file 5). Of these genes, the data from 25 (74 % of 34) matched the RNA-Seq data. As determined from the published flavonoid pathways [23], catechin biosynthesis occurs via successive enzymatic reactions (Fig. 4a). Interestingly, PAL (phenylalanine ammonia-lyase) and CHI (chalcone isomerase) genes, which are employed in the upstream phenylpropanoid pathway, were more highly expressed in oil tea than in tea. However, in the downstream biosynthetic pathway of catechins, the F3H (flavanone 3-hydroxylas), DFR (dihydroflavonol 4-reductase) and ANR (anthocyanidin reductase) genes were more highly expressed in tea. Notably, the ANR gene encodes an enzyme that catalyzes the transfer of anthocyanidins to 2,3-cis-flavan-3-ol, which is an intermediate in the final step of esterified catechin synthesis. Both RNA-Seq and qRT-PCR analyses revealed considerable activation of the ANR gene in tea but not in oil tea, which is consistent with the data from HPLC analyses of EC, EGC, C and GC contents. The DFR, LAR and ANR genes in tea are responsible for the biosynthesis of nongalloylated catechins [24]. The differential expression levels of F3H, DFR and ANR genes might be responsible for the differences detected in the levels catechin components between tea and oil tea.

Tea buds and leaves contain theanine at levels as much as 252-fold and 86-fold those of oil tea (Fig. 1), respectively. However, we did not identify genes encoding the enzyme responsible for the final reaction in theanine biosynthesis. The qRT-PCR analysis revealed that the GS (glutamine synthetase) and GDH (glutamate dehydrogenase) genes were more highly expressed in tea than in oil tea (Fig. 4b). Previous studies suggest that theanine is synthesized from glutamic acid and ethylamine by TS (theanine synthetase), which is highly homologous to glutamine GS [25]. Phytochemical analysis revealed a much higher content of theanine in tea buds and leaves than in oil tea, suggesting a potential connection between the activation of GS genes and high theanine levels in tea. In our transcriptomic data, five GS unigenes were found in tea and seven in oil tea. Whether they are functional copies of TS genes remains to be confirmed by further analysis of enzymatic reactions.

There are three key enzymes in the caffeine biosynthesis pathway: TCS (tea caffeine synthase), IMPDH (inosine-5′-monophosphate dehydrogenase) and SAMS (S-adenosylmethionine synthetase) [26]. We detected homologous genes that are involved in four steps of the caffeine pathway. TCS catalyzes the final step in caffeine biosynthesis. The TCS gene was much more highly expressed in tea buds and leaves (by over 45-fold) than in oil tea, although the genes responsible for the upstream reactions had higher transcription levels in oil tea, which was confirmed by qRT-PCR (Fig. 4c).

Taken together, our investigation of gene expression in tea revealed the activation of related metabolic pathways compared to oil tea. Most genes exhibited slightly higher expression levels in buds than in leaves (Table 4). These findings are potentially related to the differences in metabolic components revealed by HPLC.

## Discussion

In this study, we observed differences in the contents and gene expression patterns of the characteristic compounds in tea compared to oil tea. We found that tea contains more beneficial nutrients, such as catechins, theanine and caffeine, in its buds and leaves because the pathways related to these metabolites were considerably more active in tea than in oil tea. Theanine is a unique non-protein amino acid that was first discovered in tea. There are trace amounts of this compound in two other *Camellia* species (*C. japonica* and *C. sasanqua*) and in one species of mushroom (*Xerocomus badius)* [27].

Of the phenolic compounds, high flavonoid levels are present in oil tea, as revealed by HPLC (140.06 mg/g dry material) [28]. Flavonoids are a class of important secondary metabolites including flavanones, flavones, dihydroflavonols, flavonols and flavan-3-ols (catechins). These compounds are important for tea quality and are beneficial for human health (especially catechins) [29]. Catechins, theanine and caffeine are the main characteristic compounds in tea, and the results of our analysis of these compounds are in accordance with recent reports [30, 31]. Oil tea is genetically closely to tea, but no theanine and caffeine were reported except flavonoids in oil tea leaves in previous study [32, 33]. We chose tea and oil tea buds and leaves of plants from the same environment for analysis to reveal the mechanism behind the high levels catechins, theanine and caffeine in tea. Our results indicated that the catechins, theanine and caffeine in tea were also present in oil tea, but in much lower amounts. We detected increased expression of some key genes in these three metabolic pathways in tea compared to oil tea, which might lead to the differences in their contents.

Our results indicated that the genes encoding F3H, DFR and ANR in the flavonoid pathway were more highly expressed in tea than in oil tea. On the contrary, the expression levels of PAL and CHI genes were lower in tea than in oil tea. These observations were consistent with previous results [34]. High PAL activity was associated with the accumulation of flavonoids and other phenolic compounds [35, 36], and DFR, ANR and LAR played an important role in the formation of catechins [3]. Xiong *et al.* found that stable expression of F3H insured the formation of dihydrokaempferol, the precursor of individual catechins [37]. In the current study, we did not observe a difference in the expression levels of the C4H gene between tea and oil tea.

Our analysis of the DEGs related to flavonoid, theanine and caffeine metabolism in tea and oil tea suggests that these two species share common pathways, but the expression levels of some key genes in these pathyways might result in differential biosynthesis of catechins, theanine and caffeine.

Since tea is self-incompatible and recalcitrant to genetic manipulation, little genetic or genomic information is currently available for this species. Therefore, instead of providing a comprehensive in-depth investigation of the tea transcriptome, our experiment was designed to generate a quick view of the landscape. Moreover, since there were significant differences in the contents of the major components from one bud and five leaves of tea versus oil tea, we used the transcriptome data to search for key genes in these metabolic pathways and to uncover the factors underlying this divergence. The quality of tea in large part depends on its metabolic profiles. We therefore performed additional analyses of catechin, theanine and caffeine biosynthesis. We were able to detect almost all genes in these metabolic pathways. Many of these genes appeared to form multigene families, implying that the tea genome, like the genomes of many other higher plants, had undergone one or more rounds of genome duplication during evolution [38], which might explain why higher levels of gene expression did not always lead to higher enzyme activity in the present study. In our annotated tea and oil tea transcriptome dataset, multiple transcripts encoding all DEGs involved in flavonoid, theanine and caffeine biosynthesis pathways were identified.

**Fig. 5:**
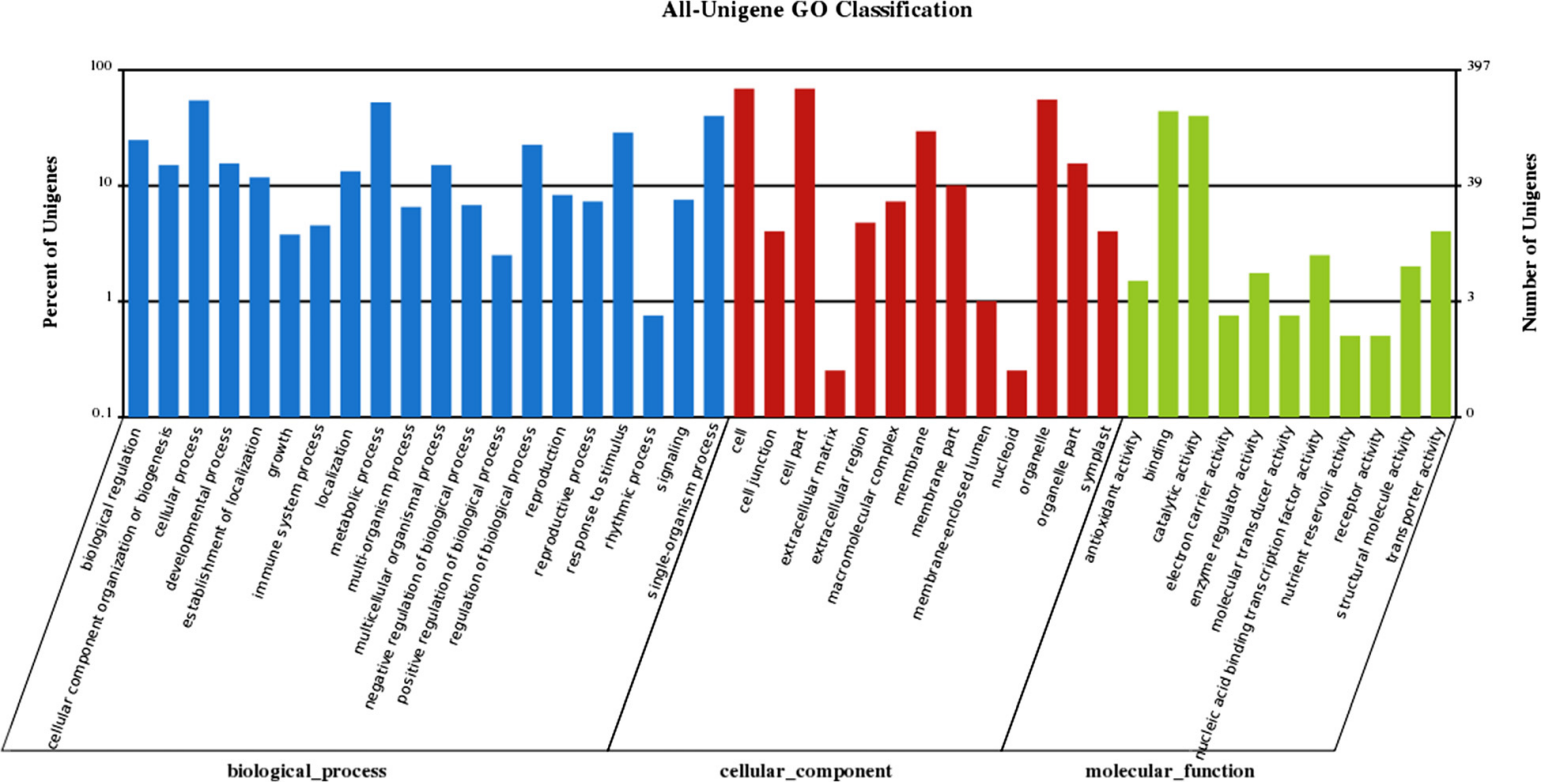
GO classification of orthologs between tea and oil tea under positive selection (Ka/Ks > 1). GO analysis of orthologous genes between tea and oil tea under positive selection based on biological process, cellular component and molecular function categories. The y-axis indicates the number of genes assigned to the same GO terms

Using a reciprocal best hit (RBH) method with relatively strict filters, 13,025 putative ortholog pairs were identified between tea and oil tea. We calculated their Ka (non-synonymous) /Ks (synonymous) ratios to estimate the rate of gene evolution [39, 40]. Of these ortholog pairs, 12,400 (95.2 % of 13,025) had a Ka/Ks value of 1 or less than 1, while 625 (4.8 % of 13,025) had a Ka/Ks value of over 1 (Additional file 6), suggesting that they were under positive selection (PS). Functional GO analysis revealed that most genes under PS were grouped into GO terms cell, cell part, binding and metabolic process (Fig. 5). Of the 625 PS genes, 68 exhibited differential expression among tissues (Additional file 7). Notably, some PS orthologs encode CHI and DFR in the flavonoid pathway. CHI is a rate-limiting enzyme, and DFR is key enzyme, in the catechin-producing branch of the flavonoid biosynthesis pathway [41, 42]. Since the Ka/Ks ratio is widely used to detect selective pressure acting on protein-coding sequences [43, 44], rapid evolution of the CHI and DFR genes might be associated with adaptive selection in plants. No PS ortholog was assigned to the theanine or caffeine pathway. Environmental factors might play an important role in the evolution of the flavonoid pathway. Indeed, the highest quality green tea from Japan (a fine powder made from tencha) was grown in the shade and contains high levels of amino acids but low levels of catechins [45].

## Conclusions

In this study, we examined the levels of characteristic metabolites in tea compared to oil tea, revealing (for the first time) trace amounts of theanine in oil tea. The contents of major metabolites were higher in tea than in oil tea. The genes involved in most of these pathways were more highly expressed in tea than in oil tea, especially key enzymes that function at branch points in these pathways, which might explain the differential biosynthesis of metabolites (resulting in different components) in tea versus oil tea. Comparative transcriptome analyses demonstrated the connection between gene expression and the biosynthesis of catechins, theanine and caffeine. Comparative transcriptome analyses comparing the levels of metabolites between tea and oil tea not only enabled us to provide a preliminary description of the gene expression profiles, but it also helped elucidate the molecular mechanisms underlying the biosynthesis of characteristic biochemicals in tea. The transcriptome data obtained in this study will serve as an invaluable platform for further studies of the molecular biology and genomes of tea and oil tea.

## Methods

### Plant materials

The six-year-old tea plants (*Camellia sinensis* [L.] O. Kuntze) and oil tea plants (*Camellia oleifera* Abel*.*) used in this study were grown in De Chang fabrication base in Anhui, China. One bud and five leaves were collected from each plant in the summer of 2013 (Fig. 1).

### Extraction and HPLC analysis of catechins, theanine and caffeine

Catechins and caffeine were extracted from the samples according to the method described by Shan *et al.* [46] with minor modifications. Briefly, 0.1 g of freeze-dried tea leaf tissue was ground in liquid nitrogen with a mortar and pestle and extracted with 3 mL 80 % methanol in an ultrasonic sonicator for 10 min at 4 °C. After centrifugation at 6,000 rpm for 10 min, the residues were re-extracted twice as described above. The supernatants were combined and diluted with 80 % methanol to a volume of 10 mL. The obtained supernatants were filtered through a 0.22 μm organic membrane before HPLC analysis.

The catechin and caffeine contents in the extracts were measured using a Waters 2695 HPLC system equipped with a 2489 ultraviolet (UV)-visible detector. A reverse-phase C18 column (Phenomenex 250 mm × 4.6 mm, 5 micron) was used at a flow rate of 1.0 mL/min. The detection wavelength was set to 278 nm, and the column temperature was 25 °C. The mobile phase consisted of 0.17 % (v/v) acetic acid (A) in water, 100 % acetonitrile (B), and the gradient elution was as follows: B 6 % from 0 to 4 min, to 14 % at 16 min, to 15 % at 22 min, to 18 % at 32 min, to 29 % at 37 min, to 45 % at 45 min, to 45 % at 50 min, to 6 % at 51 min and to 6 % at 60 min. Then, 10 μL of the filtrate was injected into the HPLC system for analysis. The filtered sample (10 μL) was injected into the HPLC system for analysis. Samples from each stage of leaf development were analyzed in triplicate.

Amino acids were extracted with hot water [47, 48]. Specifically, 0.15 g of freeze-dried tea leaves was ground in liquid nitrogen with a mortar pestle and extracted with 5 mL deionized water for 20 min in a water bath at 100 °C. After centrifugation at 6,000 rpm for 10 min, the residues were re-extracted once as described above. The supernatants were combined and diluted with water to a volume of 10 mL. The supernatants were also filtered through a 0.22 μm membrane before HPLC analysis. Theanine in tea was detected using a Waters 600E series HPLC system equipped with a quaternary pump and a 2489 ultraviolet (UV)-visible detector. A reverse-phase C18 column (Phenomenex 250 mm × 4.6 mm, 5 micron) was used at a flow rate of 1.0 mL/min. The column oven temperature was set to 25 °C. The detection wavelength was set to 199 nm for analysis [49]. The mobile phase consisted of 0.05 % (v/v) trichloroacetic acid (A) in water, 50 % acetonitrile (B), and the gradient elution was as follows: B 0 % (v/v) to 100 % at 40 min, to 100 % at 45 min and to 0 % at 60 min [31]. Then, 5 μL of the filtrate was injected into the HPLC system for analysis.

Amino acids in tea were detected using a Waters 600E series HPLC system equipped with a quaternary pump, a 2475 fluorescence detector and a 2489 ultraviolet (UV)-visible detector. The Waters AccQ•Tag method [50] with a Waters AccQ•Tag column (Nova-Pak C18, 4 μm, 150 mm × 3.9 mm) was employed to detect various amino acids according to the protocol of the AccQ•Fluor Reagent Kit [51, 52]. To determine the linearity of the chromatographic techniques, calibration plots of standards were constructed based on peak areas (y) using solutions of various concentrations (x). All plots were linear in the examined ranges; the linear ranges for different concentrations of standard compounds are shown in the plots (μg mL^−1^). The R^2^ value refers to the correlation coefficient of the equation for calculating the content of a compound. The standard compounds C, EC, EGC, ECG, EGCG, GC, theanine and caffeine were purchased from Shanghai Winherb Medical Technology, Ltd., China.

Anthocyanin was extracted as follows: 0.1 g freeze-dry tea leaf tissue was ground in liquid nitrogen and extracted with 5 mL extraction solution (80 % methanol: 1 % hydrochloric acid [HCl]) using an ultrasonic sonicator for 10 min at room temperature. After centrifugation at 6,000 rpm for 10 min, the residues were re-extracted twice as described above. The supernatants were combined and diluted with extraction solution to 10 mL, followed by extraction with trichloromethane. The anthocyanin content was determined by colorimetry at 525 nm [53].

### RNA extraction, library construction and RNA-Seq

Total RNA from tea and oil tea was extracted separately using the modified CTAB method [54]. The RNA integrity was measured using gel electrophoresis and spectrophotometry (Nanodrop). Equal amounts of RNA from three biological replicates were pooled prior to cDNA preparation. Enrichment of mRNA, fragment interruption, addition of adapters, size selection, PCR amplification and RNA-Seq were performed by staff at Beijing Genome Institute (BGI; Shenzhen, China). First, mRNA was enriched from 20 μg total RNA using magnetic beads with Oligo (dT) 25 (Invitrogen) and cleaved into short fragments. Second, using these short fragments as templates, first-strand cDNA synthesis was carried out with random primers (Japan, Takara) to produce double-stranded cDNA. Third, the ends of double-stranded cDNA fragments were further modified with T4 DNA polymerase, Klenow DNA polymerase and T4 polynucleotide kinase (Britain, NEB), and adapters were ligated to the short fragments using T4 DNA ligase (Invitrogen, USA). After the end repair process and ligation of adapters, the products were enriched by PCR to construct the final cDNA library. The cDNA library was examined using an Agilent 2100 Bioanalyzer. Finally, the four libraries were sequenced on an Illumina HiSeq™ 2000.

### De novo assembly of RNA-Seq reads

Clean reads from four samples were obtained after quality control. Of these, two were from tea and two were from oil tea, which were combined and assembled separately using the transcriptome assembler Trinity [55]. The total and average lengths of assembled contigs were important criteria for transcriptome quality. Unigenes were defined after removing redundancy and short contigs from the assembly. Unigenes from tea and oil tea were aligned to each other iteratively using BLAST to identify homologous genes in the two species; more than 80 % of the length of each gene in a pair of homologous genes was strictly aligned.

### qRT-PCR analysis of the selected genes

To validate the accuracy of unigenes obtained from the assembled transcriptome and profiling of gene expression via RNA-Seq, qRT-PCR analysis was performed. RNA samples were extracted from the samples, and single-stranded cDNAs used for real-time PCR analysis were synthesized from the RNAs using a Prime-Script™ 1st Strand cDNA Synthesis Kit (TaKaRa, Dalian, China). The expression patterns of 34 transcripts were monitored. Detailed information about the selected transcripts, including their unigene IDs and the primer pairs designed in this study, is presented in Additional file 8. An IQ5 real-time PCR detection system (Bio-Rad) was utilized as previously described. The glyceraldehyde-3-phosphate dehydrogenase (*GAPDH*) gene was used as an internal reference gene, and relative expression was calculated using the 2^ΔCt^ method [56]. All qRT-PCR analyses were performed in three biological and three technical replications.

### Unigene functional annotation and classification

The unigenes were aligned to the protein sequence database NR, the Swiss-Prot protein database and COG [57] by Blastx with an E-value threshold of 1 × 10^−5^. The unigenes were mapped to the KEGG metabolic pathway database [58]. Using KEGG annotation, metabolic pathway annotations of unigenes can be obtained, which helps elucidate the complex biological behaviors of genes. Using the COG database, orthologous gene products can be classified, and the possible functions of unigenes can be predicted. Based on NR annotation, GO classifications of unigenes were obtained using WEGO software [59] (http://wego.genomics.org.cn/cgi-bin/wego/index.pl) after annotation by the Blast 2 GO program (Version 2.3.4) [60] to elucidate the distribution of gene functions of a species at the macro level.

### Comparison of nucleotide and protein sequence in tea and oil tea

Protein sequences from tea and oil tea were compared by BLAST and MUMmer (http://mummer.sourceforge.net/), and sequences with homology ≥70 % were retained.

### Differentially expressed genes related to major secondary metabolism

KEGG pathway analysis was carried out to identify genes with different expression levels. Unigene expression was calculated using the FPKM method. The identification of differentially expressed genes (DEGs) was performed according to “The significance of digital gene expression profiles” [61], which was modified using a rigorous algorithm. FDR ≤ 0.001 and the absolute value of log_2_Ratio ≥ 1 were chosen as the thresholds for judging the significance of differential expression of each gene. For a given unigene, four FPKM values were generated from the four transcriptomes, respectively. Hierarchical clustering was performed using Cluster 3. DEGs that may play important roles in major secondary metabolism (catechins, theanine and caffeine metabolism) were identified by ggplot2 (http://docs.ggplot2.org/current/geom_point.html) [62]. After further investigating metabolic pathways, several representative pathways were selected for more detailed analyses, including flavonoid metabolism, theanine metabolism and caffeine metabolism.

### Identification of orthologous genes between tea and oil tea

To identify genes that are putatively orthologous between tea and oil tea, a RBH [63] method based on the Blastn program was used. Similar methods and criteria have been used in previous studies [64, 65]. Furthermore, GO classifications of putative orthologs under negative selection (Ka/Ks < 1) and under positive selection (Ka/Ks > 1) were compared using WEGO.

### Estimation of synonymous and non-synonymous substitution rates between orthologous gene pairs

To calculate Ks and Ka substitution rate for each orthologous gene pair, the equivalent of a biological measurement of the nonsynonymous to synonymous substitution ratio Ka/Ks [66] was introduced, in which Ks and Ka were estimated by codeml (of the PAML package) using the likelihood method [67, 68].

### Availability of supporting data

The Illumina RNA-seq data generated from buds and leaves of *Camellia sinensis* and *Camellia oleifera* are available in the NCBI SRA (http://trace.ncbi.nlm.nih.gov/Traces/sra) with accessions SRR1928149 and SRR1928150.

## Additional files

Additional file 1:
**Annotation of assembled unigenes of tea and oil tea.** (XLSX 16149 kb) Additional file 2:
**Statistical comparison of DEGs between any two tissues.** The genes were classified into three classes: red indicates up-regulated genes, green indicates down-regulated genes and blue indicates genes that are not differentially expressed. Tea buds, second leaves of tea, oil tea buds and second leaves of oil tea are abbreviated as TB, TL, OTB and OTL, respectively. (TIFF 2076 kb) Additional file 3:
**The rich factors and number of genes involved in each pathway derived from a comparison of gene expression in TBvsTL, OTBvsOTL, TBvsOTB and TL2vsOTL2.** TBvsOTB stands for tea buds versus oil tea buds, TBvsTL for tea buds versus tea leaves, TLvsOTL for tea leaves versus oil tea leaves and OTBvsOTL for oil tea buds versus leaves. (XLSX 16 kb) Additional file 4:
**Information about the genes involved in the flavonoid, theanine and caffeine biosynthesis pathways.** Unigenes involved in three metabolic pathways were annotated by alignment to the Swiss-Prot, COG and KEGG databases with e-values less than 1 × 10^−30^. (XLSX 82 kb) Additional file 5:
**List of DEGs analyzed by qRT-PCR.** A total of 34 genes involved in the flavonoid, theanine and caffeine biosynthesis pathways were analyzed by qRT-PCR. (XLSX 16 kb) Additional file 6:
**Estimated Ka and Ks values of 625 orthologous gene pairs (Ka/Ks > 1).** The sequence length, nonsynonymous substitution rate (Ka), synonymous substitution rate (Ks), Ka/Ks and P-value are shown. (XLSX 51 kb) Additional file 7:
**Distribution of DEGs under positive selection in annotated pathways.** (XLSX 12 kb) Additional file 8:
**Primers used for qRT-PCR.** (DOCX 18 kb)

## Abbreviations

HPLC: High-performance liquid chromatography
qRT-PCR: Quantitative real-time -PCR
EC: Epicatechin
GC: Gallocatechin
EGC: Epigallocatechin
C: Catechin
EGCG: Eepigallocatechin gallate
ECG: Epicatechin gallate
FPKM: Fragment Per Kilobase of exon model per Million mapped reads
NR: Non-redundant protein database
NT: Non-redundant nucleotide database
Swiss-Prot: Annotated protein sequence database
KEGG: Kyoto encyclopedia of genes and genomes
COG: Clusters of orthologous groups of protein
GO: Gene ontology
DEGs: Differentially expressed genes
TB: Tea buds
OTB: Oil tea buds
TL: Tea leaves
OTL: Oil tea leaves
PAL: Phenylalanine ammonia-lyase
CHI: Chalcone isomerase
F3H: Flavanone 3-hydroxylas
F3'5'H: Flavonoid 3',5'-hydroxylase
DFR: Dihydroflavonol 4-reductase
ANR: Anthocyanidin reductase
GS: Glutamine synthetase
GDH: Glutamate dehydrogenase
TS: Theanine synthetase
TCS: Tea caffeine synthase
IMPDH: Inosine-5′-monophosphate dehydrogenase
SAMS: S-adenosylmethionine synthetase
RBH: Reciprocal best hit
Ka: Non-synonymous
Ks: Synonymous
PS: Positive selection
HCl: Hydrochloric acid
GAPDH: Glyceraldehyde-3-phosphate dehydrogenase
