# Supplementary figures and images for: Transcriptomic and phytochemical analysis of the biosynthesis of characteristic constituents in tea (Camellia sinensis) compared with oil tea (Camellia oleifera)
Source: BMC Plant Biol. 2015 Aug 7;15:190. doi: 10.1186/s12870-015-0574-6 (PMC4527363; doi:10.1186/s12870-015-0574-6)

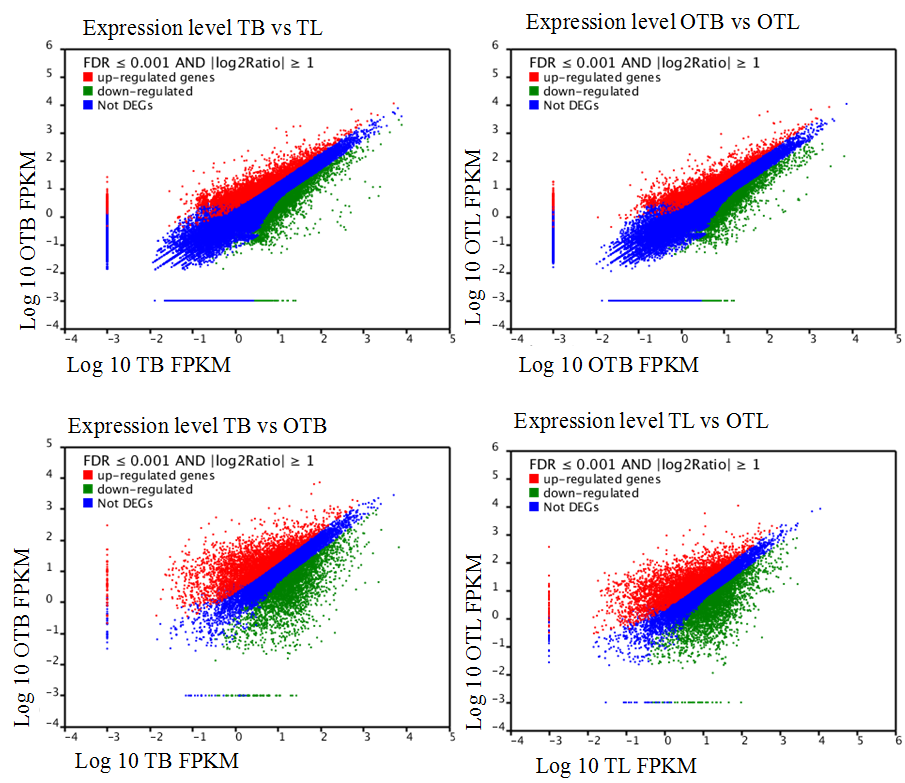

Supplement: Additional file 2: — Statistical comparison of DEGs between any two tissues. The genes were classified into three classes: red indicates up-regulated genes, green indicates down-regulated genes and blue indicates genes that are not differentially expressed. Tea buds, second leaves of tea, oil tea buds and second leaves of oil tea are abbreviated as TB, TL, OTB and OTL, respectively. (TIFF 2076 kb) [file 12870_2015_574_MOESM2_ESM.tif]
