# Supplementary material for: Transcriptomic and phytochemical analysis of the biosynthesis of characteristic constituents in tea (Camellia sinensis) compared with oil tea (Camellia oleifera)
Source: BMC Plant Biol. 2015 Aug 7;15:190. doi: 10.1186/s12870-015-0574-6 (PMC4527363; doi:10.1186/s12870-015-0574-6)
Supplement: Additional file 8: — Primers used for qRT-PCR. (DOCX 18 kb) [file 12870_2015_574_MOESM8_ESM.docx]

Primers used for qRT-PCR.

| Species | Genes | Gene ID | | Forward (5'to 3') Reverse (5' to 3') | |
| --- | --- | --- | --- | --- | --- |
| Tea | DFR | | CsiCL5790.Contig1 | GAGGTAATCAAGCCGACAATCAA | ATACATCCAGCCAGTCATCTTCT |
| Tea | FLS | | CsiUnigene16358 | AGGATGTTAAGGATGTTGTGAAGG | ACAAGGAGTCCATCAGTAGCAT |
| Tea | F3'5'H | | CsiUnigene29566 | TAGCGGAGATGTTGTGTTATGC | CCACCATGTCCTTGAACTCATT |
| Tea | 4CL | | CsiUnigene10981 | GATATTGCTGATGCTGCTGTTATAC | CACTCGCCTGATTCTCTTATATGG |
| Tea | 5'-Nase | | CsiUnigene1980 | GAGGACTCAGTTATGTCACAAGAA | GAAGTAAGCCTGGCAATCTATCTC |
| Tea | GDH | | CsiCL7628.Contig4 | GGCTCAACTAATGACCTGGAAG | ACTCACTCTTACTTAACTCACTTGG |
| Tea | GOGAT | | CsiUnigene283 | GAGGCGTGATGTATGTAACTGATA | GGAACCTGCTAAACCACAATAAAC |
| Tea | GS | | CsiCL11137.Contig3 | ACCAACTACAGTACCAAGTCCAT | CCATAAGCAGCAATGTGTTCCT |
| Tea | ALT | | CsiCL9150.Contig1 | CGAGTCCTACGAGTCTTATTATGC | GAGATACATTGCTCCTTCTGCTT |
| Tea | F3H | | CsiUnigene18521 | ATGTCTGCTCTCACTTTACTTGTC | CACTCTTGTACTTACCATTGCTTAG |
| Tea | ANR | | CsiCL5790.Contig1 | GACCTCTCACCTTCTAGCACTAA | GAACATTGACTACTCCTTGAATTGC |
| Tea | LAR | | CsiCL7884.Contig1 | GTGTTGTCAAGGATCAAGCATTC | R:GGTGAGTTGGTCTAGTATATTAGCA |
| Tea | PAL | | CsiUnigene27087 | GCTCATGTCATCTACCTACTTGG | ACTTGGCTTACTGTGTTCCTCA |
| Tea | CHI | | CsiCL7015.Contig2 | GCTGAATAAGTATTGGCATAGTTGG | TAGATTATGAAGCAGAGGCAGAAC |
| Tea | CHS | | CsiUnigene24189 | GGCATCTCTGATTGGAACTCTT | GACATATTACCGTACTCACTTAGCA |
| Tea | TCS | | CsiCL8587.Contig1 | CTCCTGTTGTAAGAGAAGCCTAC | CATTGCCTACCACGAAGTATCA |
| Tea | IMPDH | | CsiUnigene13304 | ATCATACGCTCCGCTAAGTCTC | AGATATAGTGACGACGCCTAAGAG |
| Tea | RBK | | CsiUnigene12164 | CCATCCAATCCAACCATGTCTT | GAATTGCGGACCTTAATAGGAGAT |
| Oil tea | DFR | | ColUnigene1840 | GAAGAGAATGTACCTGAGGAAGTC | GTCCAGTGGCTCTCATCAATTATG |
| Oil tea | FLS | | ColUnigene28039l | TTTGTTGAAGCTGATAGAGAGGAG | ATGGAAGAGGAGAAGAAGATGAATG |
| Oil tea | F3'5'H | | ColCL10204.Contig1 | GAGCATAACTCTCATTACACTCTCA | CACACCATAACAATCGGATACATAG |
| Oil tea | 4CL | | ColCL7952.Contig2 | AAGAATCCGATGGTGGTCACTT | ACGCCAGGCACATTGATAATAC |
| Oil tea | 5'-Nase | | ColUnigene18095 | GAGGACTCAGTTATGTCACAAGAA | GAAGTAAGCCTGGCAATCTATCTC |
| Oil tea | GDH | | ColUnigene218 | AAGACCGCAGTAGCCAATATAC | CATATCAGGTGCTGGAACATCA |
| Oil tea | GOGAT | | ColUnigene22967 | AGAAGGATGCTAGTGGGAAGTTT | GTGTTGATTCAGGACCGAGGAA |
| Oil tea | GS | | ColCL598.Contig1 | GAGAAGGTGATTGCTGAGTACATAT | GCTAGATCCGTCATAATTCCACTT |
| Oil tea | ALT | | ColCL5230.Contig1 | CGAGTCCTACGAGTCTTATTATGC | GAGATACATTGCTCCTTCTGCTT |
| Oil tea | F3H | | ColCL9855.Contig1 | GCCAATGAAGTGTATGCTGAGA | AATCAAGTCATCACCACCTACAG |
| Oil tea | ANR | | ColCL11235.Contig2 | TTGAGTTGTTGGTGTAAGTGTGAC | TGGTTGTAAGTGAGTGAGTAGTTTC |
| Oil tea | LAR | | ColCL4785.Contig3 | AGCAGCAGCAGCGAATATAATCC | TCATCAACGGTCCTGAACGATTC |
| Oil tea | PAL | | ColCL1326.Contig2 | ATCAACAACGATGACACCAAGAAC | GATTTCCACTCTCCAAAGCACTTC |
| Oil tea | CHI | | ColUnigene16344 | TTGGCAGCAAGAATGTCAGAAT | TAGATTACGAAGCAGAGGAAGAAC |
| Oil tea | CHS | | ColUnigene20161l | AGACGGAGTTGAAGGAGAAGTT | TCTAGTGAAGGTGCCATGTAGG |
| Oil tea | TCS | | ColUnigene23833 | CTCCTGTTGTAAGAGAAGCCTAC | CATTGCCTACCACGAAGTATCA |
| Oil tea | IMPDH | | ColUnigene25348l | TTCCTCTCCGATTTGACTTTCCTA | ATCAGATATAGTGACGACGCCTAA |
| Oil tea | RBK | | ColUnigene16732 | GCTCAAGTCATTATTGCTACGAAGA | AAGTGGCAGCGGAACTACTAAT |
|  | GAPDH | |  | TTGGCATCGTTGAGGGTCT | CAGTGGGAACACGGAAAGC |

Abbreviation: DFR: dihydroflavonol 4-reductase; FLS：flavonol synthase; F3'5'H: flavonoid 3',5'-hydroxylase; 4CL :4-coumarate-CoA ligase5'-Nase: 5'-nucleotidase; GDH: glutamate dehydrogenase; GOGAT: glutamate synthase; GS (glutamine synthetase); ALT (alanine aminotransferase); F3H (flavanone 3-hydroxylase); ANR (anthocyanidin reductase); LAR (leucoanthocyanidin reductase ) PAL: phenylalanine ammonia-lyase; CHI (chalcone isomerase); CHS (chalcone synthase); TCS (tea caffeine synthase); IMPDH: IMP dehydrogenase; RBK: ribokinase
